# Stable Epigenetic Programming of Effector and Central Memory CD4 T Cells Occurs Within 7 Days of Antigen Exposure *In Vivo*

**DOI:** 10.3389/fimmu.2021.642807

**Published:** 2021-05-24

**Authors:** Sarah L. Bevington, Remi Fiancette, Dominika W. Gajdasik, Peter Keane, Jake K. Soley, Claire M. Willis, Daniel J. L. Coleman, David R. Withers, Peter N. Cockerill

**Affiliations:** ^1^ Institute of Cancer and Genomic Sciences, College of Medical and Dental Sciences, University of Birmingham, Birmingham, United Kingdom; ^2^ Institute of Immunology and Immunotherapy, College of Medical and Dental Sciences, University of Birmingham, Birmingham, United Kingdom

**Keywords:** memory T CD4+ cells, gene regulatory networks, epigenetics (chromatin remodelling), immunological memory responses, T cell activation

## Abstract

T cell immunological memory is established within days of an infection, but little is known about the *in vivo* changes in gene regulatory networks accounting for their ability to respond more efficiently to secondary infections. To decipher the timing and nature of immunological memory we performed genome-wide analyses of epigenetic and transcriptional changes in a mouse model generating antigen-specific T cells. Epigenetic reprogramming for Th differentiation and memory T cell formation was already established by the peak of the T cell response after 7 days. The Th memory T cell program was associated with a gain of open chromatin regions, enriched for RUNX, ETS and T-bet motifs, which remained stable for 56 days. The epigenetic programs for both effector memory, associated with T-bet, and central memory, associated with TCF-1, were established in parallel. Memory T cell-specific regulatory elements were associated with greatly enhanced inducible Th1-biased responses during secondary exposures to antigen. Furthermore, memory T cells responded *in vivo* to re-exposure to antigen by rapidly reprograming the entire ETS factor gene regulatory network, by suppressing *Ets1* and activating *Etv6* expression. These data show that gene regulatory networks are epigenetically reprogrammed towards memory during infection, and undergo substantial changes upon re-stimulation.

## Introduction

During primary immune responses, effector T cells arise from naïve T cells (TN) when their antigen (Ag) -specific TCRs recognize Ag on Ag presenting cells (APCs) for the first time and undergo transformation over a 1-2 day period to become rapidly dividing T blast cells (TB) (1). During this process, the combination of TCR, CD28 and IL-2 signaling promotes the extensive reprograming of the T cell gene regulatory network, rendering immune response genes much more receptive to reactivation and enabling further differentiation to specialized Th cells (2–5). Systemic bacterial or viral infections *in vivo* typically establish a Th1-polarizing environment whereby additional cytokines such as IL-12 induce expression of the Th1-lineage-defining transcription factor (TF) T-bet, and T-bet target genes such as *Ifng* (IFN-γ), which reinforce Th1 status (6–8).

Secondary immune responses are dependent upon pools of long lived Ag-specific memory T cells (TM) that remain as a reservoir to fight future infections long after primary infections have resolved (9). In the absence of TCR signaling, long-term TM cells are dependent upon the homeostatic cytokines IL-2, IL-7 and/or IL-15, which each signal *via* a common gamma chain (10–12). CD4 and CD8 TM cells have retained their reprogramed status to respond much faster and more efficiently when re-exposed to Ag than naive T cells (TN) (3, 13–17). *In vitro* studies demonstrated that T cell memory is stable for at least 60 d in the absence of Ag (18). TM cells can be subdivided into two major subtypes based on their surface markers, gene expression profiles, and the nature of their responses to secondary infections (19, 20). Effector memory T cells (Tem) retain many of the properties of differentiated T cells and rapidly resume their original programed effector T cell response when reactivated. Tem cells are rapidly recruited during secondary infections and they maintain expression of differentiation inducing genes, such as *Tbx21* (T-bet) in Th1-type Tem cells. In contrast, central memory T cells (Tcm) have yet to undergo effector T cell differentiation and remain plastic and receptive to following different paths of T cell differentiation when reactivated. Thus, the transcriptional networks in Tcm cells are closer to TN cells and follicular helper T cells (Tfh), expressing factors such as *Bach2* (21–23), which represses the AP-1 responses downstream of TCR signaling (24), and *Bcl6* which polarizes cells towards Tcm or Tfh (25, 26). CD4 Tem and Tcm can also be differentiated on the basis of expression of the chemokine receptors CCR7 and CXCR5, enabling Tcm to circulate through lymphoid tissues. Tem lack expression of both CCR7 and CXCR5 and instead express receptors that enable them to traffic through non-lymphoid tissues (25–28).

The reprograming of the gene regulatory network in TB and TM cells is associated with the stable acquisition of thousands of highly accessible active chromatin regions defined as DNase I Hypersensitive Sites (DHSs) that maintain a long term transcriptional memory of their previous activation (2, 3, 29). Once formed during TB-transformation, these epigenetically primed DHSs (pDHSs) are maintained by cooperation between constitutively expressed TFs such as ETS1 and RUNX1 (2, 3), and IL-2/IL-7-inducible TFs such as STAT5 and JUND (4, 5). pDHSs play a critical role in maintaining immunological memory by maintaining broad active chromatin domains marked by the active chromatin modifications histone H3 K4me2 and H3 K27ac. These epigenetically primed domains typically encompass the inducible enhancers and promoters that mediate reactivation of immune response genes during secondary responses (2, 3).

Our understanding of CD4 TM populations has been significantly advanced by studies tracking antigen-specific responses *in vivo* (30), and in particular a model Th1 infection using an attenuated strain of *Listeria monocytogenes* (Lm) expressing the highly immunogenic peptide 2W1S (6, 26). In this acute infection, TM cells biased towards Th1 are established in lymphoid organs within 3-4 d of infection, and the T cell response reaches a peak after 6-7 d, before contracting ~7-10-fold by day 20 and then gradually subsiding further over the following months (6, 26). Interestingly, these studies demonstrated that CXCR5-ve Tem and CXCR5+ve Tcm and Tfh were established in parallel, with 90% of the CXCR5+ve cells resembling Tcm cells (26). Furthermore, the level of CXCR5 expression negatively correlated with *Il2ra* expression. *Il2ra*-deficient T cells were able to efficiently generate CXCR5^+^ PD-1^-^ Tcm cells, but they only produced 10% of the normal proportion of T-bet^high^ CXCR5^-^ Th1 cells (5, 26). Whilst the development of these memory cell populations was characterised in fine detail, the molecular basis underlying the establishment and stable maintenance of memory in Tem and Tcm cells *in vivo* was never established.

Although it is clear that the epigenetic program associated with long term immunological memory can be established within 2-3 d *in vitro*, it remains unknown how fast this is established during *in vivo* responses, or how stable it is. Most previous epigenetic analyses of TM cells formed *in vivo* have used poly-clonal populations where it has not been possible to establish how recently epigenetic memory had formed or how stable it was in long-term memory cells (3). The goal of the present study was to use the Lm-2W1S as a highly tractable *in vivo* model to study the time-course and stability of epigenetic reprograming in Ag-specific TM cells, and to investigate the role of this program in secondary Ag responses. We established that (i) the TM-epigenetic program is established in both Tcm and Tem cells within 7 days and is stable for at least 56 days, (ii) epigenetic reprograming primes immune response genes for rapid secondary responses in TM cells, and (iii) Ag-stimulation of TM cells *in vivo* leads to extensive rewiring of the ETS TF gene regulation network, with suppression of *Ets1* and ETS1-regulated genes such as *Lef1* and *Tcf7* in parallel with upregulation of the ETS family repressor *Etv6*.

## Materials and Methods

### Mice

C42 transgenic mice were used for the M7, M28 and M56 ATAC-seq and RNA-seq experiments. C42 mice have been extensively backcrossed onto a C57BL/6 background and contain a 130 kb DNA fragment of the human *IL3/CSF2* locus which can be used as a reporter of human cytokine gene activity (3). In previous studies the transgene did not have any detectable impact on the responses of these mice. WT C57BL/6 mice were used in experiments from which CXCR5+ and CXCR5- 2W1S-specific CD4 T cells were isolated. Mice were housed at 21°C +/- 2°C, 55% humidity (+/- 10%) with 12 hr light dark/cycle in 7-7 IVC caging with environmental enrichment of plastic houses plus paper bedding.

### Antigen-Specific Th Memory T Cell Generation and Purification

Mice were intravenously injected in the tail vein with 10^7^ *actA-*deficient *L. monocytogenes* expressing OVA-2W1S (*Lm*-2W1S, a kind gift from Dr. M. Jenkins) as described previously (5). To recover 2W1S-specific CD4 T cells, spleens were taken at 7, 28 or 56 days post infection, cells isolated by manual crushing of the tissue, depleted of red blood cells and then incubated with 2W1S:A^b^-APC for 1 hour at room temperature. The 2W1S-specific population was enriched as described by the Jenkins Laboratory (31) using anti-APC microbeads (Miltenyi Biotech #130-090-855). Following enrichment the cells were stained with antibodies to detect CD3, CD4, CD44, B220, CD11c and CD11b. CD44^hi^ 2W1S-specific CD4 T cells were sort purified using a BD FACSAria Fusion cell sorter (BD) to greater than 95% purity. For the re-stimulation mice were injected with 100 μg 2W1S peptide and 2.5 μg LPS 28 or 56 days post infection and purified as above 3 hours after injection. For the purification of the CXCR5 populations the cells were stained with CXCR5-PeCy7 at the same time as the 2W1S:A^b^-APC staining. Naïve CD4 T cells were purified as CD44^lo^, CD62L^hi^.

### Assay for Transposase Accessible Chromatin Using Sequencing (ATAC-Seq)

FACS purified cells were resuspended in 50 μl ATAC transposition reaction mix containing 25 μl 2x Tagment DNA Buffer (Illumina), 2.5 μl Tn5 transposase (Illumina), 0.5 µl 1% Digitonin (Promega #G9441) and incubated for 20-30 minute at 37°C with gentle agitation. For the M28Ag_1, M56Ag_1 and the CXCR5pos and CXCR5neg samples 16.5 μl PBS and 0.5 μl 10% Tween-20 were added to the transposition mix to reduce the level of background obtained in the sequencing as described by Corces et al. (32). DNA was purified using the MinElute Reaction Clean up kit (Qiagen #28204) before performing 5 cycles of PCR amplification using Nextera custom primers. The number of additional PCR cycles required to generate adequate material for sequencing was calculated using a qPCR side reaction as described (32). Amplified DNA was purified using Ampure Beads (Beckman Coulter) and libraries were validated by qPCR. Samples were sequenced on NextSeq^®^ 500/550 High Output kit v2 75 cycles (Illumina, FC 404-2005) at the Genomics Birmingham sequencing facility. Two biological replicates were sequenced for each time point.

### RNA-Sequencing (RNA-seq)

RNA was extracted using the RNeasy Plus Micro Kit (Qiagen# 74034). cDNA was generated from 0.5-3 ng of RNA using the Smart-Seq™ v4 Ultra™ Low Input RNA Kit for Sequencing (Clontech) according to the manufacturer’s instructions. Following 11 cycles of PCR amplification the number of additional cycles was calculated for each individual sample based on the quantity of cDNA which was determined using the Agilent 2100 Bioanalyzer (High Sensitivity DNA Kit #5067-4626). Libraries were prepared from 150 pg of cDNA using the Nextera XT library prep kit (Illumina FC-131-1024) and the Nextera XT index kit (Illumina FC-131-1001) according to the manufacturer’s instructions. Samples were sequenced on NextSeq^®^ 500/550 High Output Kit v2 (150 cycles) (FC-404-2002) at the Genomics Birmingham sequencing facility. Three biological replicates were sequenced for each time point.

### Data Analysis

#### Alignment, Coverage and Peak Detection of DNase-Seq, ATAC-Seq and ChIP-Seq Data Sets

Raw DNA sequencing reads were aligned to the NCBI Mouse Genome Sequencing Consortium version mm10 using *bowtie2* (Galaxy Version 2.3.2.2) (33) with the preset –very-sensitive-local. BedGraph files were generated with MACS2 callpeak (Galaxy version 2.1.1) using the default parameters (34) and were converted to bigwig files using the Wig/BedGraph-to-bigWig converter (Galaxy v1.1.1) in order to visualize on the UCSC genome browser. The statistics for ATAC-Seq read counts and peaks are shown in **Table 1**.

**Table 1:** Summary of statistics for ATAC-Seq data.

Samples	Read count	Mapped reads	Peaks
N1	55009838	50182216	30339
N2	28765208	19577334	30426
M7 1	122567768	109756779	28005
M7 2	26498877	20348656	29989
M28 1	20348656	10627634	29207
M28 2	59235548	38423698	27260
M56 1	26141267	15532278	27392
M56 2	42375252	38644631	28321
M28Ag 1	25179335	15311639	31629
M28Ag 2	55496446	47132970	36630
M56Ag 1	21467935	19320998	33110
M56Ag 2	23568973	18665342	32249
CXCR5neg_1	17313607	14127739	34792
CXCR5neg_2	16043618	15828665	32863
CXCR5pos_1	19033264	18030772	34598
CXCR5pos_2	17160597	16891953	33325

#### Normalization of ATAC-Seq Data Sets

To determine a complete set of peaks with the most accurate coordinates the BAM files from the N, M7, M28, M56, M28Ag, M56Ag ATAC-Seq samples were merged using BamTools (Galaxy Version 0.0.2) (35). MACS2 callpeak (Galaxy Version 2.1.1) was then used to generate a master set of 107467 peaks. The DNA sequence tags +/- 200 bp from the peak summit were counted for each individual sample using the annotatePeaks function of the HOMER package (36). The samples were normalized to one another using a correction factor based on the median of the top 30,000 peaks. These correction factors were then used to normalize genome browser scales, average tag density plots and contrast levels in tag density profiles.

#### Fold Change Analysis of ATAC-Seq Peaks

To generate a high confidence set of summits which could be used in downstream analyses the top 35000 peaks of the two replicate samples were intersected using BEDTools v2.26.0 intersect function (37). This generated peak groups for N, M7, M28, M56, M28Ag and M56Ag. These peaks sets were then merged to give a total of 48202 peaks which represent all the cell stages. The sequencing reads were counted using featurecounts (38) and Deseq2 (Galaxy version 2.11) (39) was used to calculate the fold change and adjusted p-values between samples. A peak was considered to be significantly differentially enriched if it had a greater than 3-fold change between samples, an adjusted p-value < 0.05 and a normalized read count >20 in both of the replicates.

#### Unions of ATAC-Seq Samples

The normalized counts from the Deseq2 analysis were used to determine a set of peaks for each sample. The peaks were filtered to only include sites with a read count >20 resulting in ~30,000 peaks/sample. The union of two samples was generated using the sort and merge function of the BEDtools package (37). The data were visualized as sequence tag density plots and ordered according to the fold change difference in tag density of one sample compared to the other.

#### DNA Sequence Tag Density Profiles

Tag density profiles were produced using the annotatePeaks function of the HOMER package (36). with -hist 10 -ghist -size 2000 as parameters. The peak summit files generated from the union of the ATAC-seq samples and the bedGraph coverage files produced by MACS2 callpeak (Galaxy Version 2.1.1) (34) were used as inputs. Images were visualized using Java Treeview (http://jtreeview.sourceforge.net/).

#### Average DNA Sequence Tag Density Plots

Average ATAC-Seq and ChIP-Seq tag density profiles were generated around the DHS summit using annotatePeaks from the HOMER package (36) with –hist 10 –size 2000 as parameters. The average sequence tag density was plotted for duplicate samples.

#### Annotation and Intersection of Peaks

The closest gene to the peak was determined using the annotatePeaks function of the HOMER package (36). Peak groups were overlapped to generate Venn diagrams using BEDtools v2.26.0 intersect function (37).

#### Motif Discovery

*De novo* DNA motif analysis was performed using the findMotifsGenome.pl function of the HOMER package (36). Motifs were identified +/- 100 bp from the peak summit.

#### Wellington Footprinting of ATAC Data to Identify Occupied TF Motifs

Aligned reads from ATAC-Seq replicates were combined to create a single bam file using the merge function in samtools v1.9 (40). These bam files were then used to plot the positions of the Tn5 integration sites on the forward and reverse strands separately using the dnase_wig_tracks.py function in Wellington, which is part of the pyDNase python package v0.3.0 (41). This tool was run in ATAC-Seq mode by specifying the -A parameter. The resulting tracks were then visualized on the UCSC genome browser (42).

Average ATAC-Seq cut profiles for individual motifs were created by first extracting the positions for each motif in the peak set being considered using the annotatePeaks.pl function in Homer v4.9.1 (36) with the options -m -mbed. The resulting bed file was then filtered to retain only motifs that were found within footprinted regions using the intersect function in bedtools v2.29.2 (37). Footprints were identified using the wellington_footprints.py function in pyDNase, which was run in ATAC-Seq mode using the options -A -fdrlimit -10. The average ATAC-Seq cut profile was then plotted around the footprinted motifs using the dnase_average_profile.py function in pyDNase using the -A parameter.

#### RNA-Seq Data Analyses

Paired-end sequence reads were processed with Trimmomatic (Galaxy version 0.38.0) (43) before alignment to the mouse genome (version mm10) using Hisat2 (Galaxy v2.1.0) (44) with default parameters. Gene expression levels were calculated with htseq-count (Galaxy version 0.9.1) (45) using RefSeq gene models as the reference transcriptome. Adjusted p-values and normalized counts were generated using Deseq2 (Galaxy version 2.11) (39). The log2 expression levels were calculated from the average of the normalized counts and the log2 FC between samples determined from these values. A gene was considered to be significantly differentially expressed if it had a greater than 3-fold change between experimental conditions, and an adjusted p-value < 0.05. The statistics for RNA-Seq read counts are shown in **Table 2**.

**Table 2:** Summary of statistics for RNA-Seq data.

Samples	Read count	Mapped reads
N1	41708821	38753290
N2	26210529	24937718
N3	40189419	38236520
M28_1	33360739	3150331
M28_2	49159947	44957133
M28_3	20466143	19511654
M56_1	21657845	20588197
M56_2	21089217	20098353
M56_3	22704067	21612454
M28Ag_1	73075749	69382406
M28Ag_2	48663357	45860315
M28Ag_3	16508204	15822670
CXCR5neg_1	32335684	28473099
CXCR5neg_2	19723427	18651578
CXCR5neg_3	31192312	29610572
CXCR5pos_1	20604093	18708539
CXCR5pos_2	43553731	41214422
CXCR5pos_3	45222585	42356577

#### KEGG Pathway Analysis

Kyoto Enyclopedia of Genes and Genomes (KEGG) pathway analysis was conducted using the ClueGO package v2.5.7 (46) in Cytoscape v3.8.2 (47). This was done using a right-sided (enrichment) test and p-values were corrected for multiple testing using the Benjamini-Hochberg method. A pathway was deemed to be significantly enriched if it had an adjusted p-value < 0.05.

#### Public Datasets

All genome-wide sequencing data generated in this study are available *via* GEO accession number GSE165348. Previously published data sets are available as follows:

ChIP-seq - TCF-1 thymocytes: GSE46662 (48).
ChIP-seq -T-BET Th1: GSE40623 (49).
ChIP-seq -JUNB TB PI (PMA+ Calcium ionophore A23187), ETS1 TB, RUNX1 TB, RUNX1 TB PI and gene expression microarray data - CD4 Naïve, Naïve PI, Memory and Memory PI: GSE67465 (3).
DNase-seq – TB IL-2, TB IL-2nil, ATAC-seq – TM Il7r^f/f,^TM CD4Cre Il7r^f/f^ and RNA-seq TB IL-2, TB IL-2nil: GSE147294 (5).

## Results

### The Memory T Cell Chromatin Signature Is Established Within 7 of a Single Episode of Acute Infection

**Figure 1:**
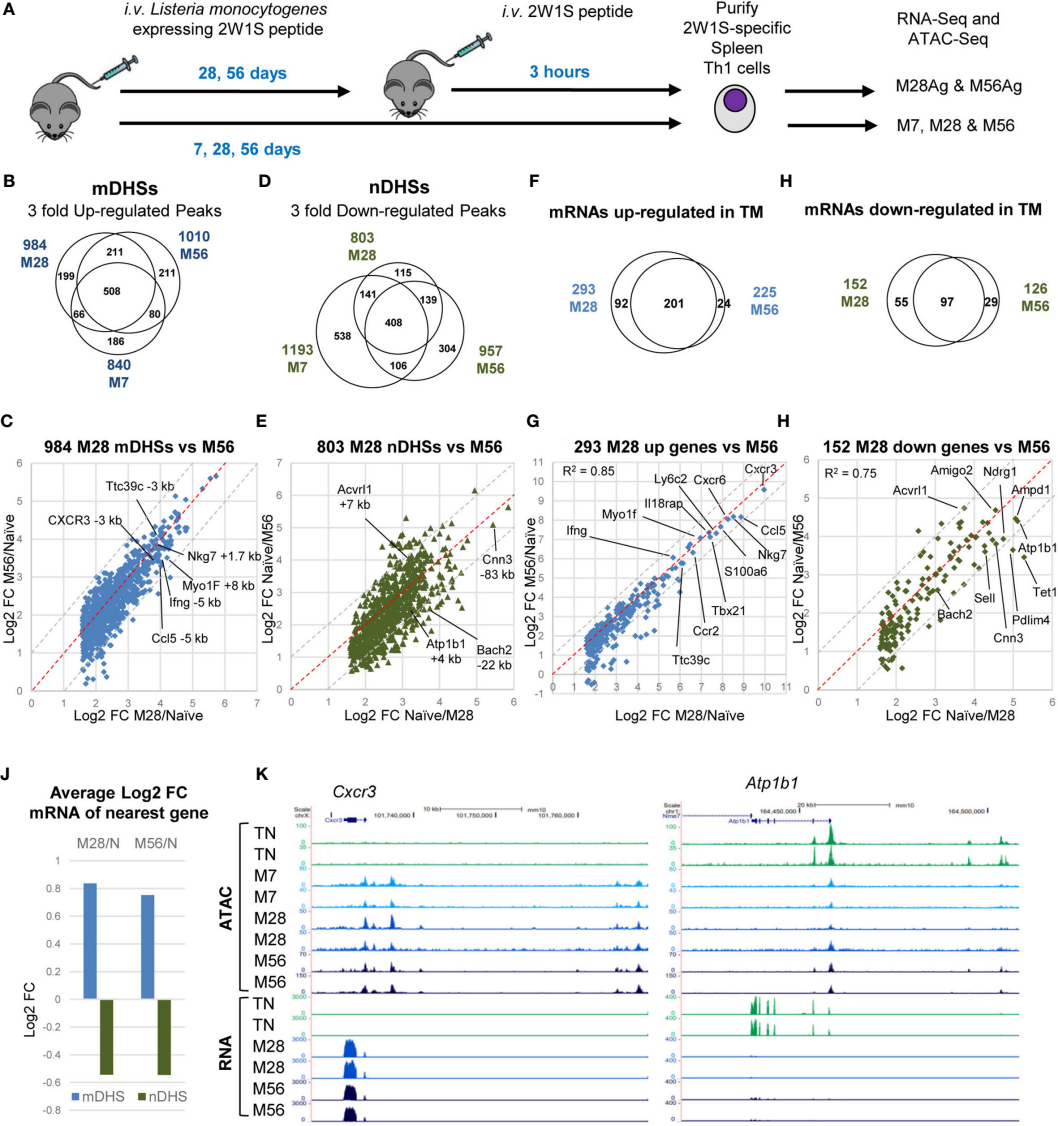
Establishment and maintenance of immunological memory in Th cells. **(A)** Protocol for the immunization of mice with Lm-2W1S. **(B–E)** Deseq2 analyses of duplicate ATAC-seq samples showing peaks which were either 3-fold up-regulated **(B, C)** or 3-fold down-regulated **(D, E)** (adj p<0.05) in M7, M28 or M56 compared to naïve CD4+, CD62L low T cells. **(F–I)** Deseq2 analyses of triplicate RNA-Seq samples showing genes which were either 3-fold up-regulated **(F, G)** or 3-fold down-regulated **(H, I)** (adj p<0.05) in M7, M28 or M56 compared to naïve CD4+, CD62L low T cells. The red dashed lines represent the equivalence points. The grey dashed lines indicate the boundaries of values that are 2-fold different. **(J)** Average log2 fold change in gene expression for genes adjacent to mDHSs or nDHSs. **(K)** UCSC browser screen shots of ATAC-seq and RNA-Seq data for the TM-specific *Cxcr3* gene and the TN-specific *Atp1b1* gene.

The aim of this study was to decipher the gene regulatory networks and chromatin signatures associated with the initial acquisition and long-term maintenance of immunological memory in 2W1S-specific TM cells, and with the recall response of TM cells re-challenged with the 2W1S peptide. We performed detailed genome-wide analyses of 2W1S-specific TM cells at the peak of the T cell response after 7 days, and then again at 28 and 56 d post-infection when the primary response and the infection had resolved and memory populations established (M7, M28 and M56), in parallel with TM cells harvested at days 28 and 56 but 3 hours after a second challenge with Ag (**Figure 1A**). Due to the tiny numbers of 2W1S-specific TN cells present in the first few days of the infection it was not possible to examine the initial Ag-inducible responses during blast cell transformation. Previous studies of BL6 mice estimated an average of just 190 2W1S-specific CD4 TN cells per mouse (31), making such analyses impractical.

To identify potential gene regulatory elements associated with immunological memory, we performed genome-wide sequencing assays of transposase-accessible chromatin (ATAC-Seq) (50). Principle component analysis of all of the ATAC data confirmed that the replicates were highly reproducible and suggested that the differences between M7, M28 and M56 were actually quite modest, with these three groups clustering closely together (**Supplementary Figure 1A**). By far the biggest differences were seen in the responses to Ag stimulation for M28Ag and M56Ag compared to non-stimulated cells.

We defined the subset of DHSs which represent immunologically primed DHSs in TM cells (mDHSs) by very rigid criteria on the basis that the average signal of the ATAC peak in M7, M28 or M56 was at least 3-fold the average value of the peak in TN with a p value of less than 0.05. In addition the peaks were filtered to have at least 20 reads in each of the 2 replicates. These analyses revealed 840 M7 ATAC peaks, 984 M28 ATAC peaks 1010 M56 ATAC peaks that were at least 3-fold greater than in TN CD4 T cells, with 508 of these mDHSs being defined by the same strict criteria in all three subsets (**Figure 1B**). Once formed at day 7, these mDHSs were remarkably persistent. Although 186 of the M7 mDHSs were not strictly defined as mDHSs in M28 and M56, they still retained a higher signal than TN at these DHSs in M28, with all except 17 DHSs being at least 2-fold higher than TN in either M28 or M56 (**Supplementary Table 1**). Furthermore, some of these transiently appearing mDHSs were detected at day 7 only by virtue of the fact that M7 cells still retained a faint activation signature from the primary infection at day 7, which subsided by day 28. For example, one weak M7-restricted mDHS located between the *Txnl4a* and *Hsbp1l1* genes was also clearly a strongly induced iDHS that recruits AP-1 and T-bet in stimulated T cells (**Supplementary Figure 2A**). KEGG pathway analysis of the genes linked to the 508 consistently identified mDHSs revealed a strong link to cytokines, cytokine and TCR signalling, and Th cell differentiation (**Supplementary Table 2**). A subset of these genes and pathways were also identified linked to the M7-specific mDHSs, including *Ifng*, *Il10*, *Il21*, and *Rora*, whereas *Il1b* and *Maf* were linked in M7 only.

By day 28, the mDHSs had largely stabilized whereby the fold increases in ATAC signals mostly remained the same within a factor of 2 at day 56 (grey dashed lines, **Figures 1B, C**). Of the 984 mDHSs detected as 3-fold higher at day 28, 820 still had an ATAC signal 3-fold higher than TN at day 56 (83%) and 959 peaks (97.5%) were still at least 2-fold higher (**Supplementary Table 1**). These highly stable mDHSs included peaks which had increased by at least 10-fold relative to TN at archetypal Th1 immune response genes including *Ifng*, *Cxcr3, Nkg7* and *Ccl5* (**Figure 1C**). The biggest differences between the three mDHS subsets were seen in the direct comparisons between the signals seen at day 28 or day 56 and the signal seen at day 7, whereby some ATAC signals continued to increase after day 7 (**Supplementary Figure 1C**). However, even then, no ATAC signals were more than 3-fold higher at either day 28 or day 56 than at day 7 (**Supplementary Table 1**).

In parallel with the establishment of mDHSs, similar numbers of TN-specific ATAC peaks (nDHSs) were lost during the acquisition of immunological memory (**Figures 1D, E** and **Supplementary Figure 1B**) at genes including *Cnn3*, *Bach2, Atp1b1* and *AcvrI1* (**Figure 1E**). The loss of sites at the *Bach2* locus is significant because it represents the suppression of a pathway that inhibits TCR-inducible AP-1 activity (24). Some of these sites continued to diminish with time, as the signals were lower in M28 and M56 than in M7 (**Supplementary Figure 1D**, **Supplementary Table 1**). In addition, 47% of the nDHSs (538/1153) which were suppressed at day 7 represented transient changes, most likely again due to the persistence of a weak activation signature at day 7, as they had increased in magnitude again by day 28 (**Figure 1D**). For example, several weak DHSs at the *Adarb2*/*Wdr37* locus were defined as nDHSs in M7, reappeared in M28 and M56, but were then suppressed by Ag in M28Ag and M56Ag (**Supplementary Figure 2B**). Two of these nDHSs bind either TCF-1 or ETS1.

To correlate the most stable changes in chromatin accessibility with changes in gene expression we performed parallel analyses of RNA-Seq data for M28 and M56 relative to TN (**Figures 1F–I** and **Supplementary Table 3**). These analyses identified consistent changes in 201 genes that were upregulated and 97 genes that were downregulated in M28 and M56 compared to TN. The magnitude of increase in mRNA values also remained remarkably consistent from day 28 to day 56 for genes including *Ifng*, *Cxcr3*, and *Ccl5*, which are known to be regulated by T-bet in Th1 cells (**Figure 1G**), and where parallel chromatin changes were observed (**Figure 1C**). The expression of the genes *Cnn3*, *Bach2, Atp1b1* and *AcvrI1* also decreased in parallel with the loss of nDHSs (**Figures 1E, I**). Strongly down-regulated genes included the archetypal TN-associated gene *Sell* encoding for L-Selectin (CD62L) that functions to localise T cells in lymph nodes (51). Globally there were substantial changes in mRNA levels for genes associated with either mDHSs or nDHSs (**Figure 1J**). Examples of ATAC-Seq and RNA-Seq data are depicted for the TM-specific *Cxcr3* gene and the TN-specific *Atp1b1* gene (**Figure 1K**).

### The Acquisition of Immunological Memory Is Associated With Changes in Gene Regulatory Networks

**Figure 2:**
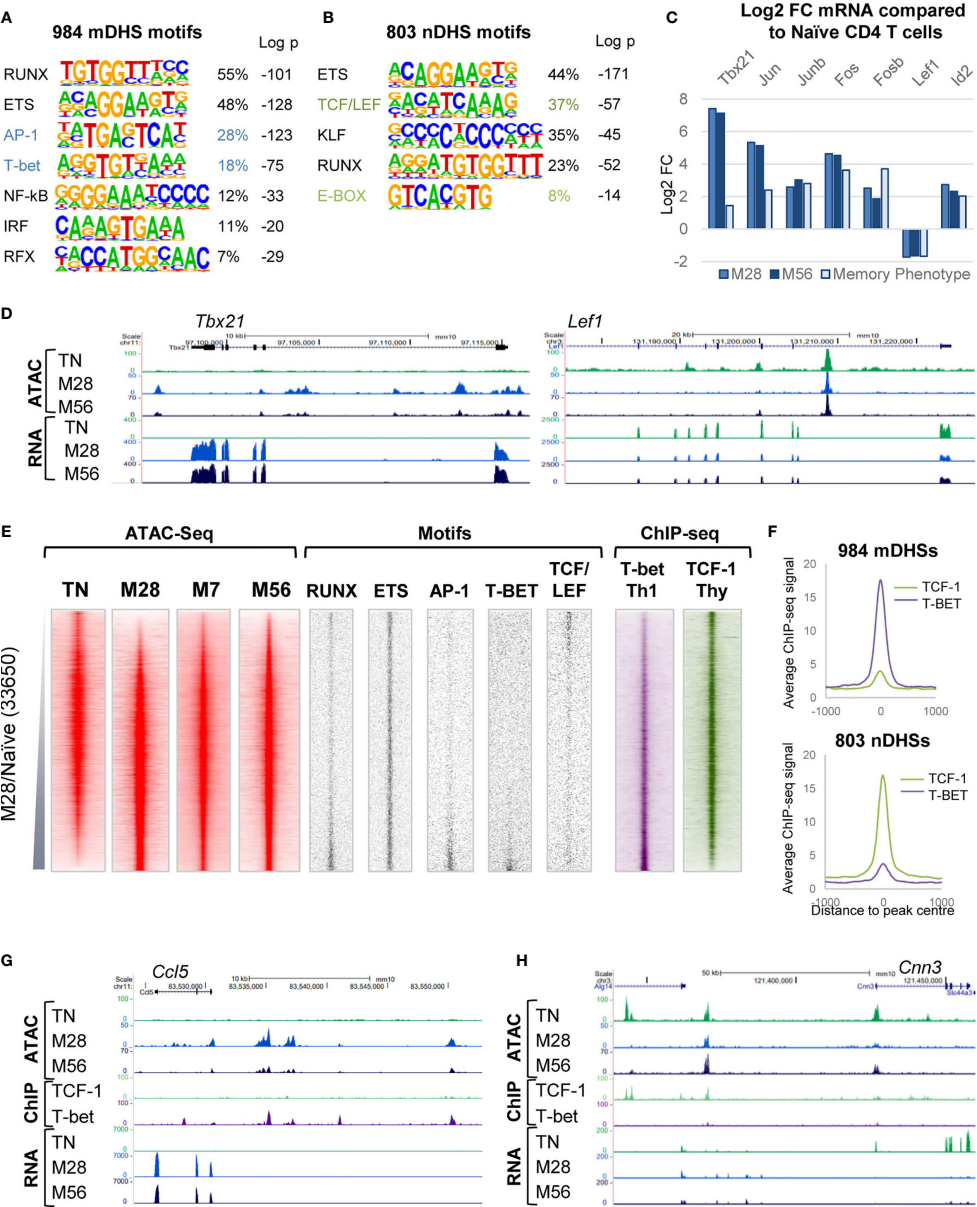
Gene regulatory networks associated with immunological memory in T cells. **(A, B)** HOMER *de novo* DNA motif analyses of TF motifs that are enriched in mDHSs that are gained **(A)** or nDHSs that are lost **(B)** during the acquisition of immunological memory. **(C)** Log2 values of the fold change (FC) in mRNA expression of TF genes associated with motifs enriched in the mDHSs and nDHSs. Data are shown for M28 or M56 TM cells relative to TN and for previously published microarray data for CD4 memory phenotype cells compared to naïve T cells (3). **(D)** UCSC browser screen shots of ATAC-seq and RNA-Seq data for the TM-specific *Tbx21* gene and the TN-specific *Lef1* gene. **(E)** Global analyses of all DHSs present in either TN or M28 TM cells (replicate 1), ranked according to fold increase in ATAC-seq signal. Shown alongside on the same coordinates are ATAC-seq signals for M7 and M56 TM cells (replicate 1), TF motifs associated with mDHSs and nDHSs, and published ChIP-Seq data for T-bet in Th1 cells (49) and TCF-1 in thymocytes (48). **(F)** Average TCF-1 and T-bet ChIP-Seq profiles for the mDHSs and nDHSs as displayed in **(E)**. **(G, H)** UCSC browser screen shots of ATAC-seq, ChIP-Seq and RNA-Seq data for the TM-specific *Ccl5* gene **(G)** and the TN-specific *Cnn3* gene **(H)**.

To investigate the underlying basis of the TM and TN gene regulatory networks we performed HOMER *de novo* DNA motif-finding analyses of the TM-specific mDHSs detected in M28 and the TN-specific nDHSs that were lost in M28 (**Figures 2A, B**). The Th-specific mDHSs were enriched for binding sites for the Th1 lineage-defining factor T-bet (*Tbx21*), and for the constitutively expressed factors ETS-1 and RUNX1 that function globally to support immunological memory in bulk TM cells (3). These sites were also enriched for the inducible TF motif for AP-1 which may be one of the factors needed to initially open up mDHSs during the acute phase of infection (3). The nDHSs were characterized by TCF/LEF motifs, consistent with a shutdown of the *Lef1*/*Tcf7* (TCF-1)-associated TN program when naïve T cells are transformed to effector T cells. Analyses of gene expression for transcription factors (TFs) linked to the differentially regulated motifs (**Figure 2C**) indicated that *Tbx21*, and the AP-1 genes *Jun*, *Junb*, *Fos* and *Fosb* were all upregulated in M28 and M56 Th memory T cells as well as in data from purified bulk naturally arising CD4 memory T cells analysed in a previous study, which had been defined as “memory phenotype” on the basis of being CD4 and CD44 positive but CD62L negative (3). The loss of the TN program was reflected by down regulation of *Lef1* and upregulation of *Id2*, an inhibitor of HLH family proteins (**Figure 2C**), which may account for the decrease in nDHSs containing E-Box motifs for HLH TFs (**Figure 2B**). These changes in mRNA were also reflected in changes in the ATAC profiles at the *Tbx21* and *Lef1* loci (**Figure 2D**).

Global analyses of ATAC and TF DNA motif profiles were performed for all DHSs present in either TN or M28, ranked according to their relative ATAC-seq signals, and displayed alongside the ATAC signals for the same DNA elements in M7 and M56 cells (**Figure 2E**). These data confirmed that the TM-specific mDHSs were enriched for RUNX, ETS, AP-1 and T-bet motifs, whereas the naïve-specific nDHSs were enriched for ETS and in TCF/LEF motifs. Parallel analyses of published chromatin immunoprecipitation (ChIP) data for T-bet in Th1 cells (49), and for TCF-1 in thymus derived naïve T cells (48), confirmed binding of T-bet to the mDHSs and TCF-1 to the nDHSs (**Figures 2E, F**). Examples of these patterns of regulation are depicted for T-bet binding to TM-specific *Ccl5* (**Figure 2G**) and for TCF-1 binding to TN-specific *Cnn3* (**Figure 2H**).

To find indirect evidence that the above motifs were potentially occupied by the predicted families of TFs, we reanalysed the ATAC data using the Wellington footprinting algorithm (41) to plot average profiles of TF motifs. For this purpose we combined the ATAC replicates and plotted the cumulative merged ATAC profiles on the forward and reverse strand sequence reads for the ATAC profiles centred over each motif, to generate average profiles for the mDHSs and nDHSs in both TM and TN (**Supplementary Figure 3**). For the 984 mDHSs, these data revealed strong protection of ETS motifs, and modest protection of AP-1, T-bet and TCF/LEF motifs in TM cells, whereas these footprints were not seen in TN cells where the DHSs were absent. For the 803 nDHSs, these data revealed strong protection of ETS and TCF/LEF motifs, and poor protection of AP-1and T-bet motifs in TN cells, whereas these footprints were not seen in TM cells where the ATAC signal was much lower.

### Lm-2W1S-Specific Memory T Cells Respond Robustly to Re-Exposure to 2W1S

**Figure 3:**
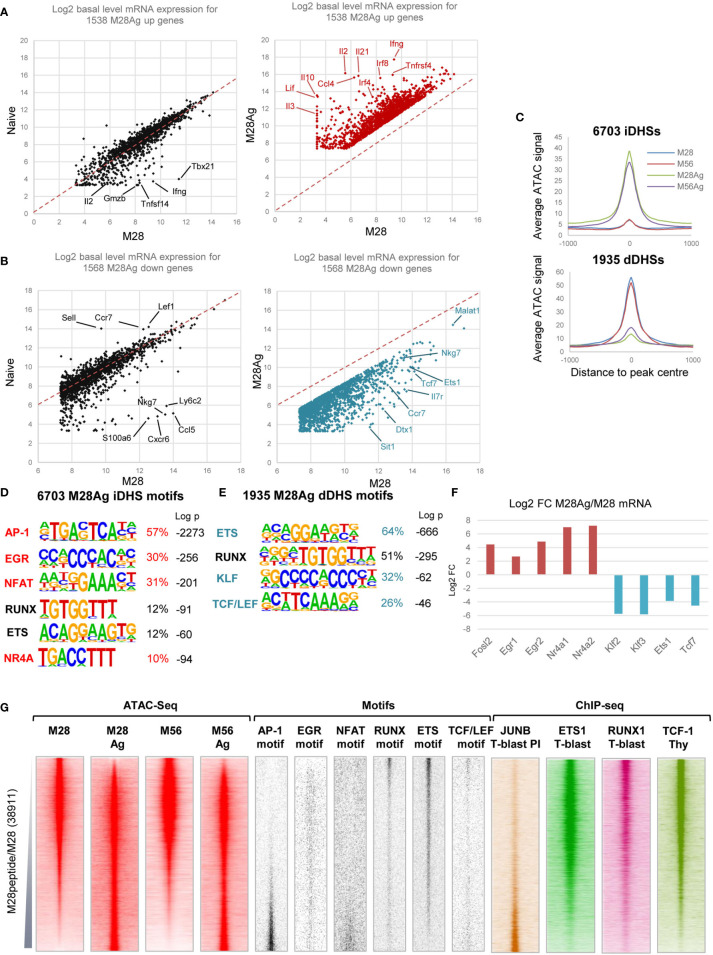
Gene regulatory networks associated with inducible genes in TM cells. **(A)** RNA-seq data for 3-fold inducible genes in M28 TM cells plotted versus Ag-stimulated M28 TM cells (M28Ag, red) or TN cells (black). **(B)** RNA-seq data for 3-fold inhibited genes in M28 TM cells plotted versus M28Ag TM cells (blue) or TN cells (black). **(C)** Average ATAC-Seq profiles for DHSs which are either 3-fold induced (iDHSs) or 3-fold diminished (dDHSs) in M28Ag. Data are shown as an average of the two replicates **(D, E)** HOMER *de novo* DNA motif analyses of TF motifs that are enriched in iDHSs **(D)** or dDHSs **(E)**. **(F)** Log2 values of the fold change (FC) in mRNA expression of TF genes associated with motifs enriched in iDHSs or dDHSs. **(G)** Global analyses of all DHSs present in either M28 TM cells or M28Ag TM cells (replicate 2), ranked according to fold increase in ATAC-seq signal. Shown alongside on the same coordinates are ATAC-seq signals for M56 and M56Ag TM cells (replicate 2), TF motifs associated with iDHSs and dDHSs, and published ChIP-Seq data for JUNB in PI-stimulated TB cells, ETS1 and RUNX1 in TB cells (3) and TCF-1 in thymocytes (48).

We next investigated the relationships between epigenetic priming in M28 and M56, and the responses of these cells to a second challenge with Ag. M28 and M56 mice were injected *i.v.* with the 2W1S peptide 3 hours prior to purification of 2W1S-specific T cells (**Figure 1A**). 1538 genes were at least 3-fold upregulated by this *in vivo* stimulation (red dots, **Figure 3A**, **Supplementary Table 3**). Strikingly, most of these inducible genes were maintained in homeostasis at the same levels in both TN and M28 in the absence of stimulation (black dots, **Figure 3A**). The exceptions to this were the 77/1538 genes (5%) that were already 3-fold upregulated in M28 relative to TN, and were then further upregulated by at least 3-fold by Ag, including genes such as *Tbx21, Gzmb, Ifng, Tnfsf14*, and *Il2* (**Figure 3A**). 30 of these genes were already associated with mDHSs prior to re-stimulation (**Supplementary Table 3**), and many of these Ag-primed genes were previously found to be non-inducible by PMA+ Calcium ionophore A23187 (PI) in TN cells where they lack priming (3). There were also 1568 genes which were at least 3-fold downregulated after a 3 hour *in vivo* exposure to 2W1S (blue dots, **Figure 3B** and **Supplementary Table 3**). These genes were also mostly maintained at the same levels of expression in TN and M28 cells under homeostatic conditions, (black dots, **Figure 3B**). Genes strongly downregulated by Ag in M28 T cells included *Tcf7, Ets1, Nkg7, Malat1, Ccr7* and *Il7r*.

Re-stimulation of TM cells with Ag induced global changes in accessible chromatin profiles. Analyses of the ATAC profiles revealed 6703 DHSs induced at day 28 (iDHSs), and 1935 diminished DHSs (dDHSs) that were rapidly suppressed by Ag at day 28 (**Figure 3C** and **Supplementary Table 1**). HOMER *de novo* DNA motif finding analyses found that the iDHSs were dominated by the archetypal inducible AP-1, NFAT, EGR and NR4A motifs classically associated with activation of TCR signalling throughout the T cell lineage (**Figure 3D**), as seen in previous studies (3, 52, 53), in combination with a lower level of RUNX and ETS motifs. The dDHSs were much more highly enriched for RUNX and ETS motifs, which maintain immunological memory at mDHSs in TM cells during homeostasis (3), in addition to KLF and TCT/LEF motifs (**Figure 3E**) which were also associated with TN-specific DHSs (**Figure 2B**). The enrichment of motifs specifically in iDHSs and dDHSs was reflected by parallel changes in mRNA levels of the associated TFs (**Figure 3F**).

Global analyses were performed for ATAC and TF DNA motif profiles of all DHSs present either before or after stimulation in M28, ranked according to their relative ATAC signals. Displayed alongside are the signals for the same DNA elements in M56 and M56Ag cells confirming that these regulatory regions were also induced at the later time point (**Figure 3G**). These data confirmed that the iDHSs were highly enriched for AP-1 and NFAT motifs, but not for RUNX, ETS or TCF/LEF motifs which were instead highly enriched in the dDHSs present in non-stimulated cells (**Figure 3G**). In contrast, the Ag-suppressed dDHSs were devoid of AP-1 motifs (**Figure 3G**), consistent with previous studies of dDHSs which disappear following *in vitro* activation of TCR signalling in T cells (2, 3). Published ChIP-Seq data also confirmed that the AP-1 factor JUNB bound to the iDHSs in T-blast cells stimulated with PI whereas TCF-1, ETS-1 and RUNX1 were enriched at the dDHSs in either TB cells or thymocytes (**Figure 3G**). The EGR motif was not, however, concentrated in the iDHSs, perhaps owing to its similarity to the widely distributed Sp1 motif which shares the sequence CCGCCC.

Here we again used Wellington to look for evidence of occupancy or loss of occupancy of the motifs of interest before and after Ag stimulation. This time we plotted average ATAC profiles for motifs in the iDHSs and dDHSs using the accumulated merged ATAC signals in all M28 and M56 samples compared to all M28Ag and M56Ag samples (**Supplementary Figure 4**). For the 6703 iDHSs, these data revealed strong protection of the inducible AP-1 and NFAT motifs, and moderate protection of ETS and TCF/LEF motifs in Ag-stimulated TM cells, with much weaker ATAC activity before stimulation. For the 1935 dDHSs, the ETS motifs showed very strong ATAC activity and footprint protection before stimulation and much less activity after stimulation. In addition, the dDHSs showed a complete absence of AP-1 footprints, and weak NFAT footprints prior to stimulation, consistent with the motif plots in **Figure 3G**. The footprints seen at TCF/LEF motifs in TM cells also diminished following stimulation. Overall, these data suggest that dDHSs are unresponsive to inducible factors but have a strong dependence on ETS factors which are no longer able to sustain them following stimulation. Due to the very low numbers of TM cells which can be purified it is technically not possible for us to perform ChIP assays to confirm loss of binding of ETS1 at the dDHSs, however the loss of open chromatin makes it highly unlikely that many TFs could remain bound at these sites.

**Figure 4:**
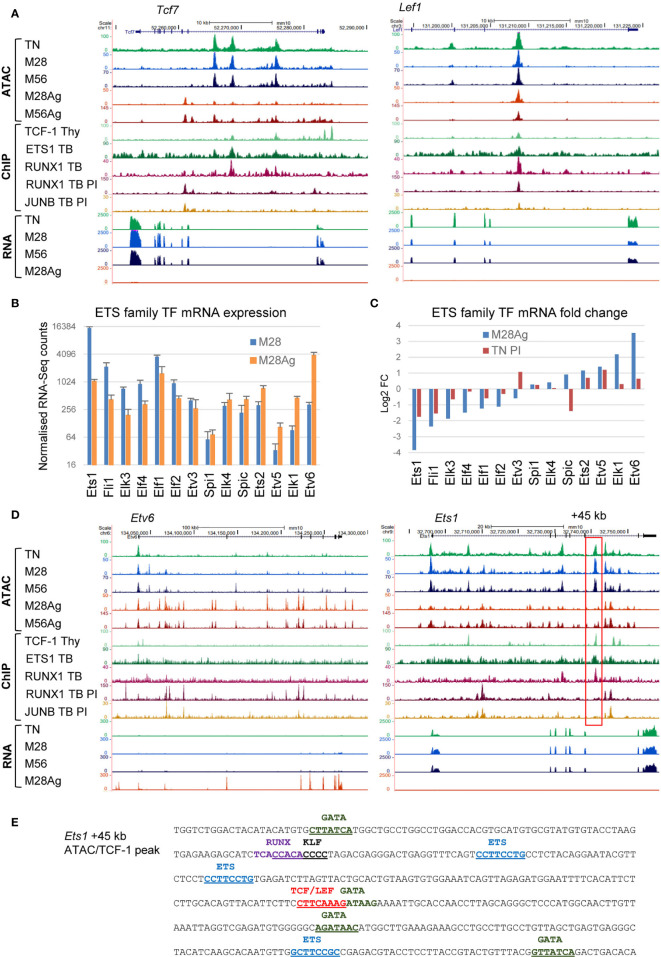
Rewiring or the ETS and TCF/LEF gene regulatory networks in response to Ag. **(A)** UCSC browser screen shots for *Tcf7* and *Lef1* showing ATAC-seq and RNA-Seq data in M28 and M56 TM cells, before or after Ag-stimulation. Also depicted are published ChIP-Seq data for TCF-1 in thymocytes (48), and ETS1, RUNX1 and JUNB, in TB cells before or after PI-stimulation (3). **(B)** RNA-seq data for all ETS family genes expressed in M28 or M28Ag. The standard deviation is shown for 3 replicates. **(C)** Log2 fold changes in gene expression for the data shown in **(B)**, in parallel with equivalent published data for naïve T cells treated with PI (3). **(D)** UCSC browser screen shots for *Etv6* and *Ets1*, the same data is depicted as for **(A)**. The red box highlights a DHS at + 45 kb which is suppressed in response to Ag. **(E)** DNA sequence of the *Ets1* +45 kb dDHS showing relevant TF motifs.

The global loss of DHSs with protected TCF/LEF and ETS motifs in response to Ag stimulation was also accompanied by substantial decreases in mRNA expression and ATAC-seq signals at the *Tcf7* and *Lef1* loci (**Figure 4A**), and striking changes in expression of most of the ETS family TFs expressed in Ag-stimulated M28 cells (**Figures 4B, C**). These observations were supported by ChIP-Seq data from TB cells cultured *in vitro* showing binding of ETS1 and RUNX1 at ATAC peaks which are reduced upon stimulation with PI or Ag (**Figure 4A**). In response to *in vivo* activation by Ag, the expression of *Ets1* was suppressed by ~15-fold, while conversely the expression of *Etv6*, a repressor of ETS activity, was induced more than 10-fold. These changes were mirrored by extensive changes in the ATAC-Seq profiles at both the *Etv6* and *Ets1* loci, which included iDHSs bound by the AP-1 TF JUNB and RUNX1 in PI-stimulated TB cells at *Etv6*, and a binding site for TCF-1, ETS1 and RUNX1 at +45 kb ATAC peak in the *Ets1* locus which disappeared upon stimulation (**Figure 4D**). This +45 kb *Ets1* peak encompassed 3 ETS motifs, TCF/LEF and KLF motifs (**Figure 4E**), suggesting that its disappearance is linked to the downregulation of TFs such as *Ets1, Fli1, Elf1, Klf2, Klf3*,*Tcf7* and *Lef1* (**Figures 2C**, **3F**, **4C**). The +45 kb DHS also had 4 ideal GATA motifs, suggesting a potential alternate mode of regulation in Th2 cells that express GATA3. The inducible changes in ETS family gene expression were not, however, limited to TM cells as similar trends were observed to a smaller degree in published RNA micro-array data for PI-stimulated TN cells (**Figure 4C**). For example, *Ets1* and *Fli1* expression is suppressed 3-4 fold by PI in TN cells. Overall, it would appear that ETS1 plays a bigger role in maintaining the homeostatic T cell program, and immunological memory at mDHSs, than in activating inducible genes in Th1 cells. This is similar to the role previously defined for the IL-2/IL-7 inducible AP-1 family member JUND, which may maintain epigenetic priming without actually activating many AP-1 target genes which have low steady state levels of mRNA in TM cells (4, 5).

### TM-Specific and Inducible DHSs Cooperate in the Memory Recall Response

**Figure 5:**
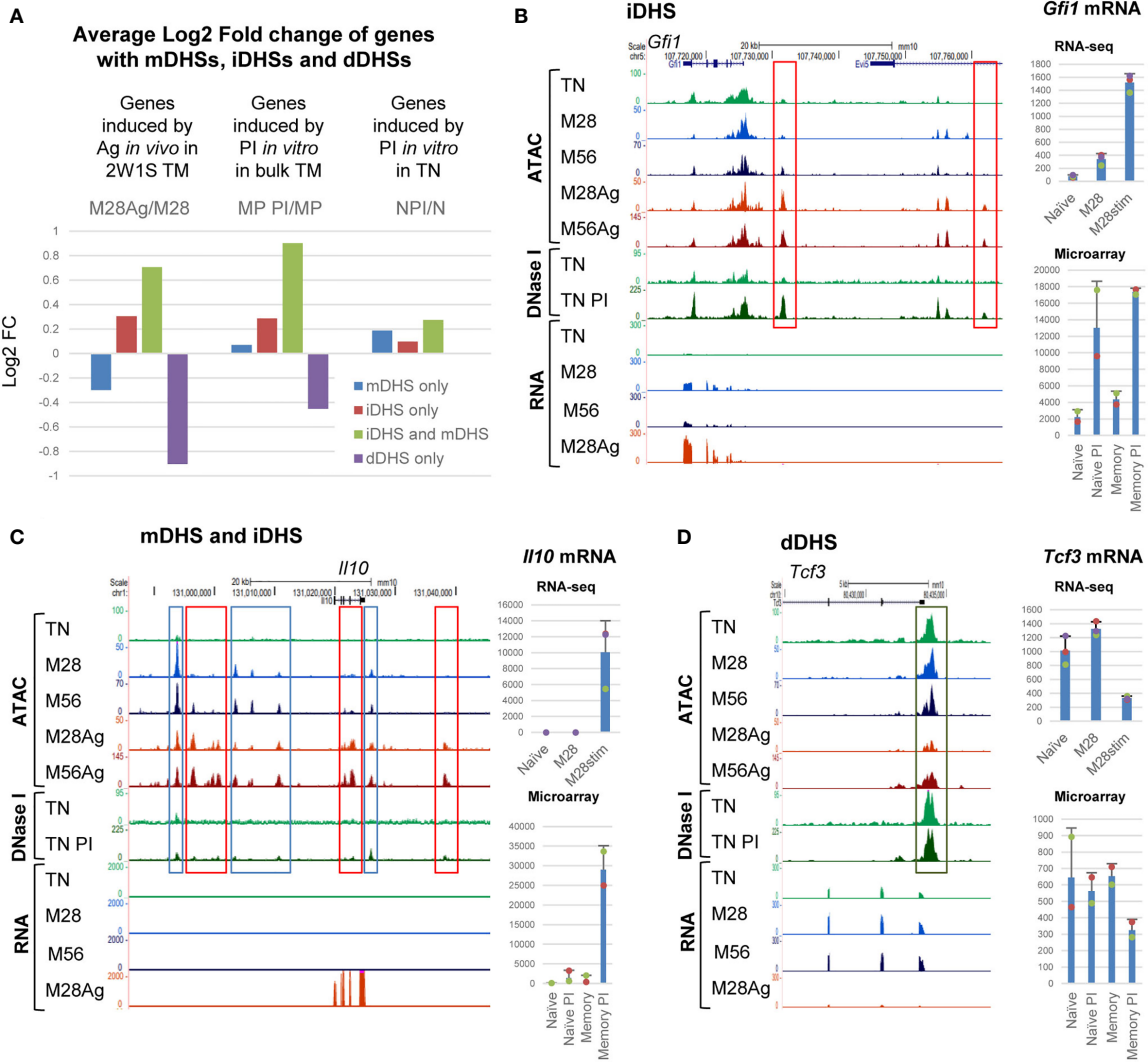
Inducible TM-specific genes have primed mDHSs associated with iDHSs. **(A)** Average Log2 values of the fold change (FC) in mRNA expression of genes associated with just mDHSs, just iDHSs, both mDHSs and iDHSs, or just dDHSs. Values are shown for mRNA changes following Ag-stimulation of M28 TM cells. Also shown are published microarray data for responses to PI-stimulation of CD4 memory phenotype cells (MP) and naïve T cells (N) (3). **(B–D)** UCSC browser screen shots showing ATAC-Seq, DNase-Seq and RNA-Seq data for representative genes with iDHSs **(B)**, mDHSs and iDHSs **(C)**, or dDHSs **(D)**. Tracks include published DNase-Seq data for naïve CD4 T cells (TN) before and after PI-stimulation (3). Red boxes highlight iDHSs, blue boxes highlight mDHSs, and the dark green box highlights a dDHS. Shown at the right are RNA-Seq data for TN, M28 and M28 Ag (top) and published microarray data for responses to PI-stimulation of CD4 memory phenotype cells and naïve T cells (bottom) (3). The standard deviation is shown for 3 replicates for the RNA-seq data and 2 replicates for the microarray data.

Previous *in vitro* studies suggested that much of the inducible gene expression program in memory T cells is dependent upon epigenetic priming of immune response genes (2, 3). Here we investigated the extent to which epigenetic reprograming was associated with the *in vivo* responses of memory T cells by looking for correlations between the inducibility of genes and their association with just mDHSs, just iDHSs, or iDHSs together with mDHSs. Global analyses of average mRNA expression revealed that genes which are associated with both iDHSs and mDHSs are more highly inducible by Ag in M28, or by PI in previously published data on bulk TM cells (3) than the other subsets (**Figure 5A**, green bars). Furthermore the same genes showed little response to stimulation by PI in TN cells which lack these mDHSs and genes with dDHSs actually went down in expression after activation. Taken together these data support the published model proposing that memory specific mDHSs aid the induction of proximal iDHSs and subsequent gene expression (2, 3). Examples of these concepts can be seen for: (i) Genes such as *Gfi1, Rel* and *Tnf*, where iDHSs appeared in both naïve and memory cells and which were induced in each of TN PI, TM PI and M28 Ag, with (**Figure 5B**, and **Supplementary Figure 5A**). These loci have pre-existing DHSs in TN and lack memory specific mDHSs accounting for the induction in both cell types. (ii) Genes such as *Il10*, *Il21* and *Ifng*, which have both mDHSs and iDHSs, showed higher levels of inducibility in M28 cells, and were PI-inducible in TM cells but not in TN cells where they lack pre-existing DHSs (**Figure 5C** and **Supplementary Figure 5B**). (iii) Genes with mDHSs only were, on average, expressed at a higher level in M28 and M56 cells than TN cells, but were not induced by Ag, and actually decreased in expression (**Supplementary Figure 5C**). On the other side of the spectrum, genes that have dDHSs which were lost after stimulation, such as *Tcf3* and *Pik3r5*, were expressed at a lower level in M28Ag than M28, but were not suppressed by PI-stimulation in TN cells (**Figures 5A, D** and **Supplementary Figure 5D**).

KEGG pathway analysis of the subsets of genes defined in **Figure 5A** consistently identified a strong link to cytokines, cytokine and TCR signalling, and Th cell differentiation in genes with mDHSs and/or iDHSs (**Supplementary Table 2**). KEGG analysis of genes with just dDHSs identified just 3 pathways, including endocytosis and apoptosis.

### The Epigenetic Programs for Central Memory and Effector Memory Are Both Established Within 7 Days of an Acute Episode of Infection

**Figure 6:**
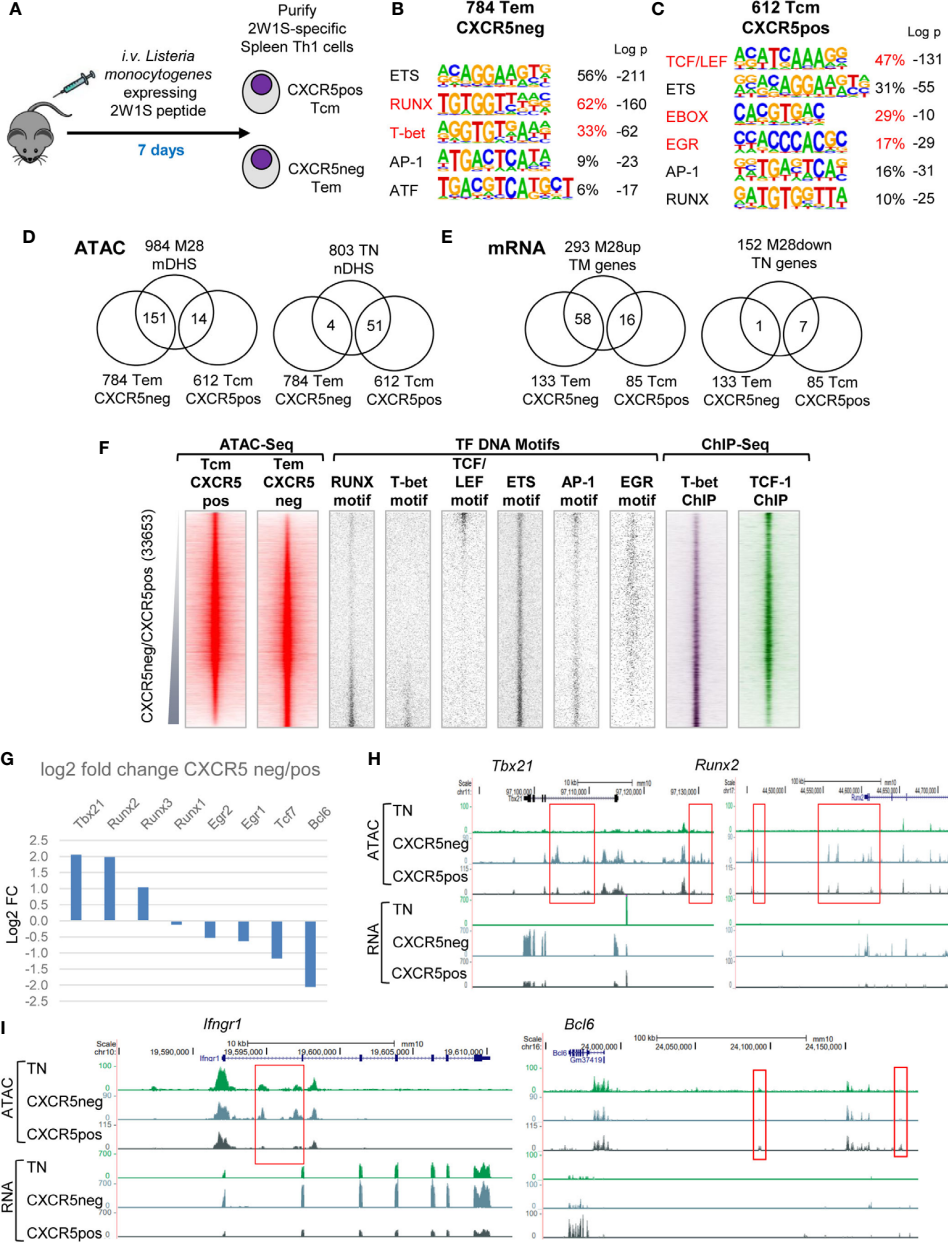
Tem and Tcm cells establish distinct chromatin and mRNA profiles. **(A)** Protocol for the immunization of mice with Lm-2W1S to generate Tem and Tcm cells. **(B, C)** HOMER *de novo* DNA motif analyses of TF motifs that are enriched in Tem **(B)** and Tcm **(C)**-specific DHSs. **(D, E)** Venn diagrams showing overlaps between Tem and Tcm-specific mDHSs identified by ATAC-Seq **(D)** and genes identified by RNA-Seq **(E)** and the corresponding TM and TN-specific DHSs and genes. **(F)** Global analyses of all DHSs present in either Tcm or Tem cells (replicate 1), ranked according to fold change in ATAC-seq signal. Shown alongside on the same coordinates are TF motifs associated with Tem and Tcm-specific DHSs, and published ChIP-Seq data for T-bet in Th1 cells (49) and TCF-1 in thymocytes (48). **(G)** Log2 values of the fold change (FC) in mRNA expression of selected differentially regulated TF genes. **(H, I)** UCSC browser screen shots showing ATAC-Seq and RNA-Seq data for TN, CXCR5-ve Tem and CXCR5+ve Tcm cells, showing representative examples of Tem-specific and Tcm-specific genes **(E)**. Red boxes highlight differentially regulated DHSs.

To investigate mechanisms involved in establishing both central and effector memory, we analysed Ag-specific TM cells soon after they had formed, 7 days after infection with Lm-2W1S, and separated 2W1S-specific CD4 T cells into CXCR5-ve Tem and CXCR5+ve Tcm cells (**Figure 6A**). Comparisons of ATAC-Seq data from these two populations identified 784 mDHSs that were 2-fold greater in Tem cells and 612 mDHSs that were 2-fold greater in Tcm cells (**Supplementary Table 4**). DNA motif analyses determined that these two subsets of DHSs had distinct gene regulation signatures. The Tem subgroup was enriched for T-bet motifs, similar the M28 mDHSs, whereas the Tcm subgroup was enriched for TCF/LEF and E-box motifs, similar to the TN-specific nDHSs (**Figures 6B, C**). However, despite these overall similarities with other DHS subsets, there was relatively little overlap between the Tem-specific and M28-specific mDHSs (151/984 mDHSs) and genes (58/293) (**Figures 6D, E** and **Supplementary Table 1**). There was even less overlap between the Tcm-specific and TN-specific DHSs (51/803 nDHSs) and genes (7/152) (**Figures 6D, E**). Hence, the split between Tem and Tcm programs is not simply a reversion of Tcm cells to a more primitive state closer to the TN gene regulatory network. What is more likely is that Tcm and TN share a part of the cell quiescence program defined by TCF-1 and LEF1, whereas recently activated Tem develop a stronger commitment to Th1 differentiation, and that these two pathways of Ag response develop in parallel. KEGG pathway analysis of the subsets of genes linked to the Tem and Tcm-specific mDHSs identified in **Figure 6D** revealed a strong link to cytokines, cytokine and TCR signalling and Th cell differentiation in both subsets, whereas genes such as *Nfkb1* and *Mapk14* also had Tem-specific mDHSs linked to pathways associated with intracellular infections that drive Th1 responses (Human immunodeficiency virus 1 infection and Tuberculosis, **Supplementary Table 2**).

More detailed global analyses of the gene regulatory networks underlying the above-defined Tem-specific and Tcm-specific DHS subsets (**Figure 6F**) further confirmed the notion that Tem-specific sites were the regulatory elements showing the strongest commitment to Th1 differentiation, being most enriched for T-bet motifs. Conversely, the Tcm-specific DHSs revealed a lack of Th1 commitment and were heavily biased to the TCF/LEF program associated with dormant TN cells or thymocytes. These patterns were reflected by the published ChIP-Seq data for T-bet from Th1 cells and TCF1 from thymocytes (**Figure 6F**). Although AP-1 and EGR family TFs are both MAPK inducible, and AP-1 motifs were enriched in both Tem and Tcm-specific DHSs, the EGR motif was found in Tcm but not Tem-specific DHSs.

Closer inspection of the TF gene expression profiles confirmed that the Th1-defining TF *Tbx21* and the Th1-inducing IFN-g receptor gene *Ifngr1* were more highly expressed in Tem, and had Tem-specific DHSs (**Figures 6G–I**). The upregulation of *Runx2* and *Runx3* expression might partially account for the enrichment of RUNX motifs in Tem-specific DHSs (**Figure 6G**). Conversely, the Tfh-associated TF gene *Bcl6* was upregulated in Tcm, but not TN or Tem, as further evidence that Tcm cells had also moved beyond the TN stage (**Figure 6I**). Bcl6 is already known to play a major role in steering recently activated T cells towards Tcm fate, and is downregulated during secondary responses when Tcm cells are recruited as effector T cells (26).

### The Choice of Cytokine Receptors Influences the Decision Between Tem and Tcm Commitment

**Figure 7:**
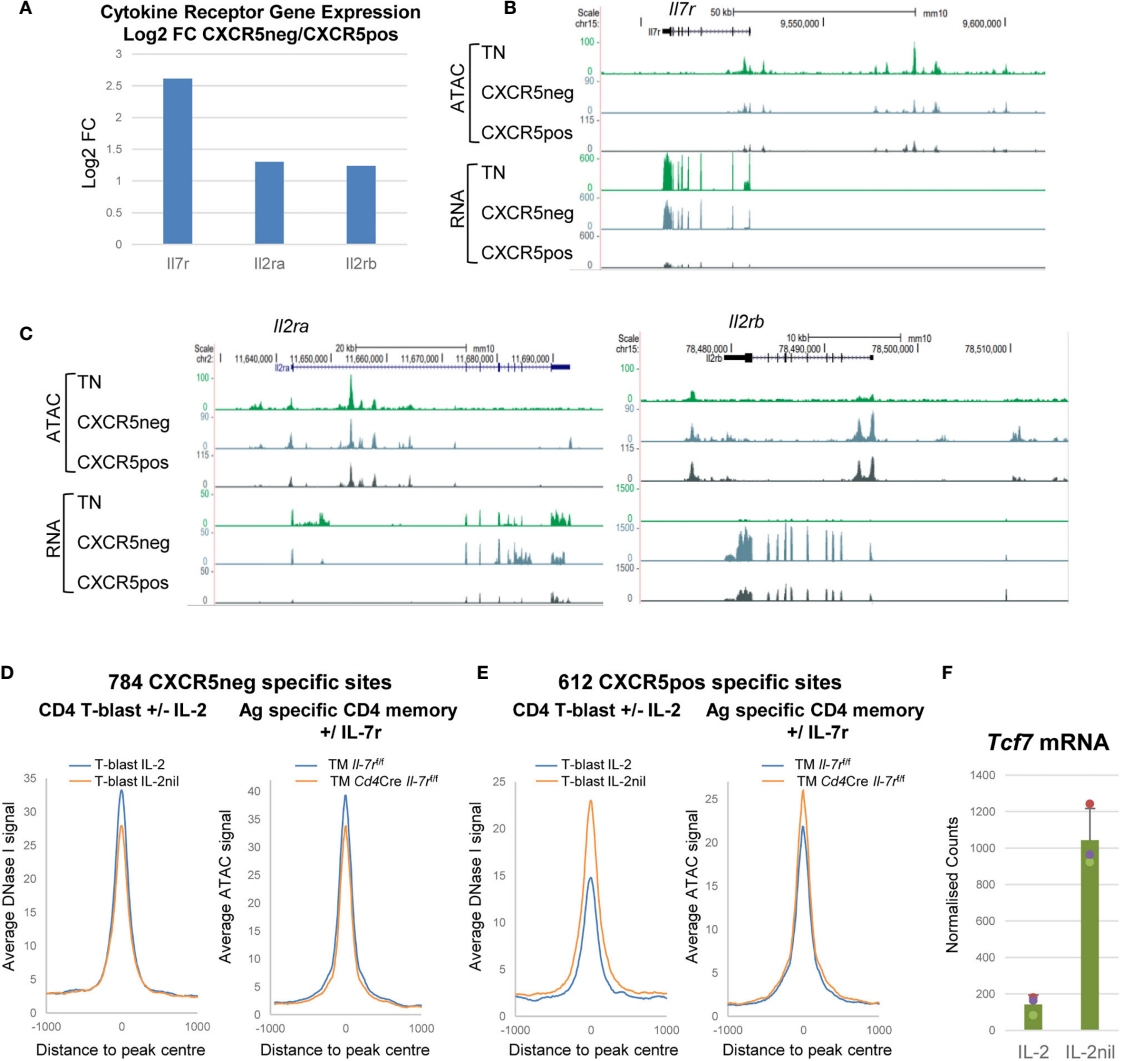
Cytokine receptor genes are differentially regulated in Tem and Tcm cells. **(A)** Log2 values of the fold change (FC) in mRNA expression of selected cytokine receptor genes in Tem relative to Tcm cells. **(B, C)** UCSC browser screen shots showing ATAC-Seq and RNA-Seq data for TN, CXCR5-ve Tem and CXCR5+ve Tcm cells, for IL-7 **(B)** and IL-2 **(B)** receptor genes. **(D, E)** Published data (5) depicting average DNase-Seq and ATAC-seq profiles for TB cells cultured in the presence or absence of IL-2 (left panels) and for Ag-specific Th TM cells before or after *in vivo* deletion of the *Il7r* gene (right panels) for Tem-specific **(D)** and Tcm-specific **(E)** DHSs. **(F)** Published mRNA-Seq data (5) for *Tcf7* expression in TB cells cultured in the presence or absence of IL-2.

Sometimes, branches in lineage commitment are decided by stochastic choices in gene expression programs at differentiation branch points. This is in essence the cornerstone of the Waddington model of the role of epigenetics in differentiation (54). This may also be what separates the choices between Tem and Tcm fate. In this model, the cell fates are determined according to which cytokine receptors a recently activated T cell expresses. In this study, Tem cells exhibit much higher expression of the *Il7r*, *IL2ra* and *Il2b* genes than Tcm cells (**Figures 7A–C**) consistent with the previous observation that Tcm cells lack IL-2Rα expression (5, 26). This is highly significant because we recently demonstrated that IL-2/IL-7 signalling *via* the common gamma chain plays a crucial role in maintaining accessible sites in lineage-specific genes prior to T cell differentiation (5). Without this priming for T cell differentiation, Tcm cells may be left in limbo, unable to differentiate, but progress towards an equally important fate of establishing and maintaining a plastic memory of TCR activation, before any lineage-commitment decision has been made. Evidence suggests that IL-2 and IL-7 play major roles in determining Tem versus Tcm fate because recently activated T cells have DHSs which are biased towards Tem fate in the presence of IL-2 or when they express the IL-7 receptor (**Figure 7D**), but Tcm fate in the absence of IL-2 or when the *Il7r* is deleted (**Figure 7E**). Furthermore, expression of *Tcf7*, encoding TCF-1, is strongly down-regulated in the presence of IL-2, supporting a model where TCF-1 maintains “stemness” and quiescence (**Figure 7F**).

## Discussion

### Immunological Memory Is Established Within 7 Days and Remains Stable

Previous *in vitro* studies suggested that the epigenetic and gene expression program underlying T cell memory is established within 2-3 d of activation of TCR signaling in naïve T cells (3, 13). This program relies on epigenetic priming of inducible genes that can be rapidly re-activated in a TM-specific manner. Mouse studies of Ag-specific memory T cells demonstrated that the mRNA expression program for immunological memory in response to acute episodes of bacterial infections is already established at the peak of the Th1 response within 7 d of infection (20). Here we used the same model system to confirm that epigenetic priming also forms the underlying basis of the acquisition of immunological memory in Th cells. Acute infection by Lm-2W1S triggered Th differentiation associated with stable expression of the Th1 lineage-defining factor T-bet and the epigenetic priming of hundreds of inducible genes. As seen in previous global studies of TM cells (20), this program was predominantly maintained by binding of the constitutively expressed TFs ETS1 and RUNX1 to sites which had previously been inaccessible in TN cells. These DNA elements also contained a high proportion of motifs for AP-1 which is thought to both support the initial opening up of mDHSs during the activation phase (3), and bind IL-2/IL-7-inducible JUND and STAT5 (5), to keep genes accessible for lineage-defining factors including T-bet during differentiation (5). Once immunological memory has been established in T cells, it is remarkably stable in the absence of further TCR activation. In rapidly dividing cells cultured with IL-2 or IL-7, mDHSs recruit TFs that include ETS1, RUNX1, JUND and STAT5 which maintain an open chromatin environment rendered more accessible by histone H3K4me2 and H3K9ac (3, 5). In the context of immune homeostasis, immunological priming of TM cells is likely to remain stable for decades in quiescent TM cells in the presence of lymph node derived IL-7 or tissue-derived IL-15 (5, 10–12).

In this study we also showed the Th1 program developed in parallel with the transcriptional and epigenetic silencing of genes regulated by a TCF/LEF-dependent network in TN cells such as *Lef1*, *Bach2*, *Sell*, *Atp1b1, Ndgr1* and *Cnn3*. These changes may reflect a shut-down of a quiescent homeostatic program in TN cells where BACH2 silences the inducible AP-1 network (24). Some of these down regulated genes are known to control cell behavior and migration whereby L-Selectin (*Sell*) facilitates migration into lymph nodes, Calponin 3 (*Cnn3*) regulates actin in the cytoskeleton (55) where it can control contraction of actin stress fibers (56), and NDRG1 functions as a metastasis suppressor and blocks signaling to NF-κB (57, 58). NDRG1 is a T cell anergy factor that is suppressed by CD28 and IL-2 signaling, and NDRG1-deficient mice show T-cell hyper-responsiveness (59). NDRG1 is also likely to help maintain the TCF/LEF network by interacting with b-Catenin (60).

### The Th Tem and Tcm Programs Develop in Parallel

One of the key questions in memory T cell biology is whether memory T cells evolve in parallel with effector T cells, or represent effector T cells that have returned to a quiescent state. Studies of the acute response to Lm2W1S suggested that these two mechanisms operate in parallel, producing both Tem cells and Tcm cells within the same time frame (26). Previous studies of Ag-specific T cells studies revealed that a systemic Lm-2W1S infection initiates a Th1-biased T cell response where (i) some TN cells differentiate as CXCR5-ve cells, expressing T-bet and CD25 (IL-2Rα), which support the Th1 program and revert to Tem memory T cells once the infection is cleared, and (ii) CXCR5+ve cells which expand in response to TCR signaling, but evade Th1 differentiation and develop as Tcm cells lacking CD25 expression (26). Here we established that (i) the Tem program is supported by a Th1-like gene regulatory network dominated by epigenetic priming maintained by T-bet, ETS and RUNX factors, and (ii) the Tcm program involves establishment of a different epigenetic program, but one maintained by a gene regulatory network involving TCF/LEF and HLH E-box-binding TFs. The choices between these two fates may well be stochastic whereby cells are biased towards either a Th1 pathway in Tem supported by IL-2 and IL-7 receptor signaling, or a Tcm pathway in cells with low IL-2 and IL-7 receptor expression and high BCL6 expression, which maintain the TCF/LEF network and thereby evade Th1 differentiation. However, in this study we did not track cell fate during the primary response, and so we have not been able formally identify the direct precursors of Tem and Tcm cells. Furthermore, a recent single cell analysis of CD4 T cell clones developing from malarial infection identified considerable heterogeneity in cell fates, and proposed that TM cells gradually arise from a pool of effector T cells rather than bypassing this stage (61). A similar study of CD8 T cells also found that viral infection leads to a heterogeneous population of responding cells that includes early arising effector T cells, that have an activation phenotype and have silenced genes associated with T cell memory (62). Following cell division, these effector T cells could subsequently generate distinct sub-populations of cells exhibiting either a Tem, Tcm or a Th1 cell phenotype, exhibiting differential expression of genes such as *Tbx21*, *Tcf7* and *Id2*.

### The Ag Recall Response Involves Extensive Rewiring of the ETS TF Network

Here we confirmed the previously proposed epigenetic priming model (2, 3) as the basis of the acquisition of immunological memory in T cells. We showed that a stable Ag recall response in *bona fide* long term Ag-specific memory T cells was indeed associated with epigenetic priming of gene regulatory elements within inducible effector T cell-specific genes. We demonstrated global epigenetic priming at DNA elements associated with greatly enhanced Ag responses in TM cells but not TN cells. In addition, a subset of these primed mDHSs were associated with genes that have increased steady state expression in Th1 cells such as *Tbx21* and *Ccl5*. In each case, the gene regulatory networks were supported by T-bet and ETS and RUNX factors.

One striking observation made here was that reactivation of TM cells by Ag resulted in a very rapid and global rewiring of the ETS gene regulatory networks. Previous studies conclusively established that ETS factors played crucial roles at every stage of hematopoietic, thymocyte and T cell development (63, 64). Early thymocyte development is associated with progressive down-regulation of the repressive ETS factor ETV6 and upregulation of the transcriptional activator ETS1 (65). ETS1 cooperates with LEF-1 and RUNX1 to activate expression of TCR genes (66–69). ETS1 is also essential for the development of a Th1 response in cells able to express IFN-γ (70). Paradoxically, we found that Ag-stimulation *in vivo* results in the almost immediate shutdown of the *Ets1* gene and activation of the *Etv6* gene. Taken together, these data suggest that TFs such as ETS1 are essential for driving T cell development and differentiation, and for maintaining immunological memory during homeostasis without activating transcription of immune response genes. In parallel with this switch in the role of ETS factors, we previously described opposing roles for different AP-1 proteins whereby (i) JUND maintains IL-2/IL-7-dependent priming during homeostasis, without activating transcription, and (ii) other TCR-inducible AP-1 proteins such as FOS, JUN and JUNB which drive reactivation of the immune response (5). Furthermore, previous studies found that depletion of *Ets1* or *JunD* in mice led to an increase in the number of activated T cells, and defects in Treg cells, suggesting that both ETS1 and JUND help to maintain homeostasis (71, 72).

In conclusion, the rewiring of the gene regulation program during T cell development, differentiation and activation involves many more levels than were previously fully appreciated. The memory T cell homeostasis program is holding genes in a poised receptive state, ready to be activated at a moment’s notice, and this program itself has to be shut down during an immune response to recall Ags.

## Data Availability Statement

The datasets presented in this study can be found in online repositories. The names of the repository/repositories and accession number(s) are: GEO, accessed via GSE165348.

## Ethics Statement

The animal study was reviewed and approved by UK Home Office.

## Author Contributions

SB, RF, DG, JS, CW, and DC performed the experiments. SB and PK analyzed the data. SB, DW, and PC wrote the manuscript. All authors contributed to the article and approved the submitted version.

## Funding

This study was supported by funding from the Medical Research Council (MR/P001319/1).

## Conflict of Interest

The authors declare that the research was conducted in the absence of any commercial or financial relationships that could be construed as a potential conflict of interest.
