# Supplementary material for: Stable Epigenetic Programming of Effector and Central Memory CD4 T Cells Occurs Within 7 Days of Antigen Exposure In Vivo
Source: Front Immunol. 2021 May 24;12:642807. doi: 10.3389/fimmu.2021.642807 (PMC8181421; doi:10.3389/fimmu.2021.642807)
Supplement: Supplementary file 1 [file DataSheet_1.pdf]

Supplementary material

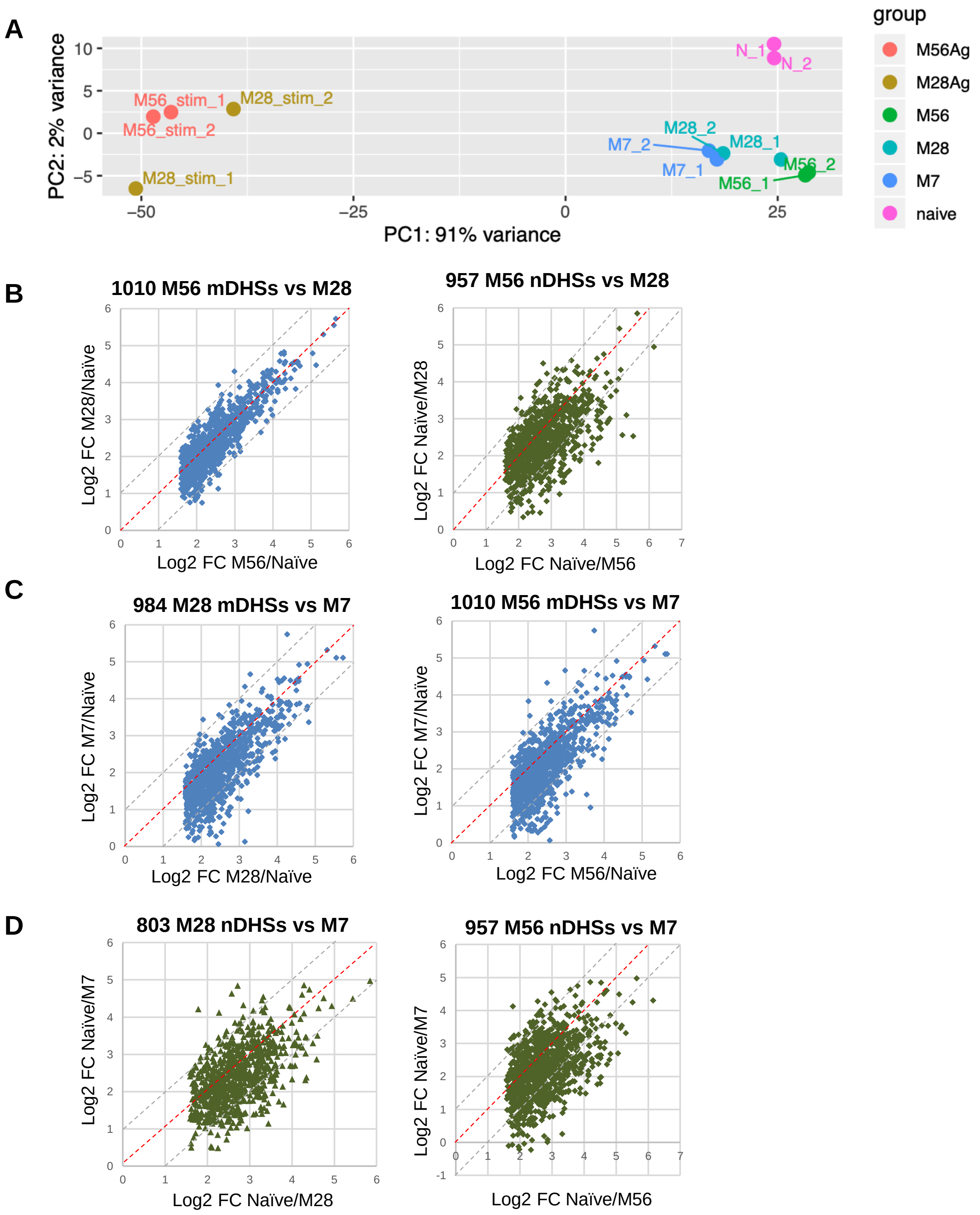

Figure S1. Long term maintenance of chromatin profiles in Th TM cells.
(**A**) Principal Component Analysis (PCA) of duplicate ATAC-Seq data from TN, M7, M28, M56, M28Ag and M56Ag. (**B-D**) Log2 fold change values for ATAC-Seq data for mDHSs and nDHSs in TM cells relative to TN cells for M28 compared to M56 (B), mDHSs in M28 and M56 relative to M7 (C), and nDHSs in M28 and M56 relative to M7 (D). The red dashed lines represent the equivalence points. The grey dashed lines indicate the boundaries of values that are 2-fold different.

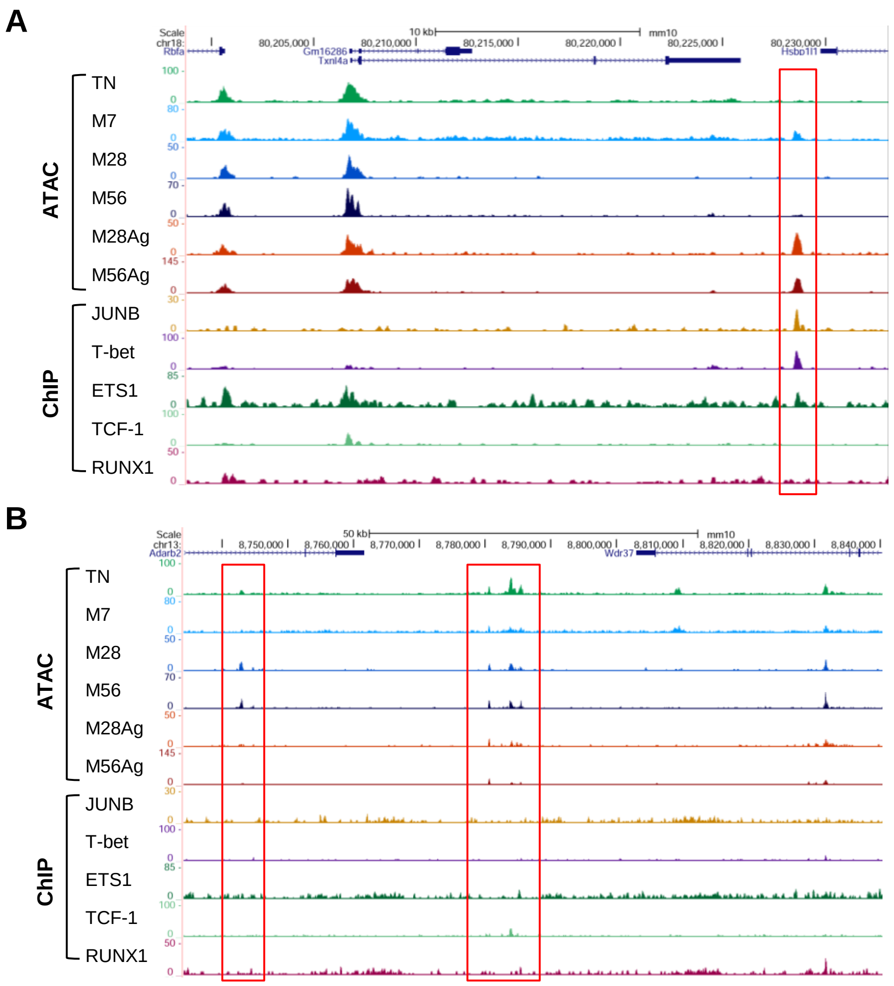

Figure S2

Examples of changes in chromatin that appear transiently 7 days post infection.

UCSC genome browser screenshots of DHSs.

(A) Example of an M7-specific mDHS, seen at day 7 but not 28 or 56, which is also an iDHS.

(B) Example of an M7-specific nDHS, seen at day 7 but not 28 or 56, which is also a dDHS.

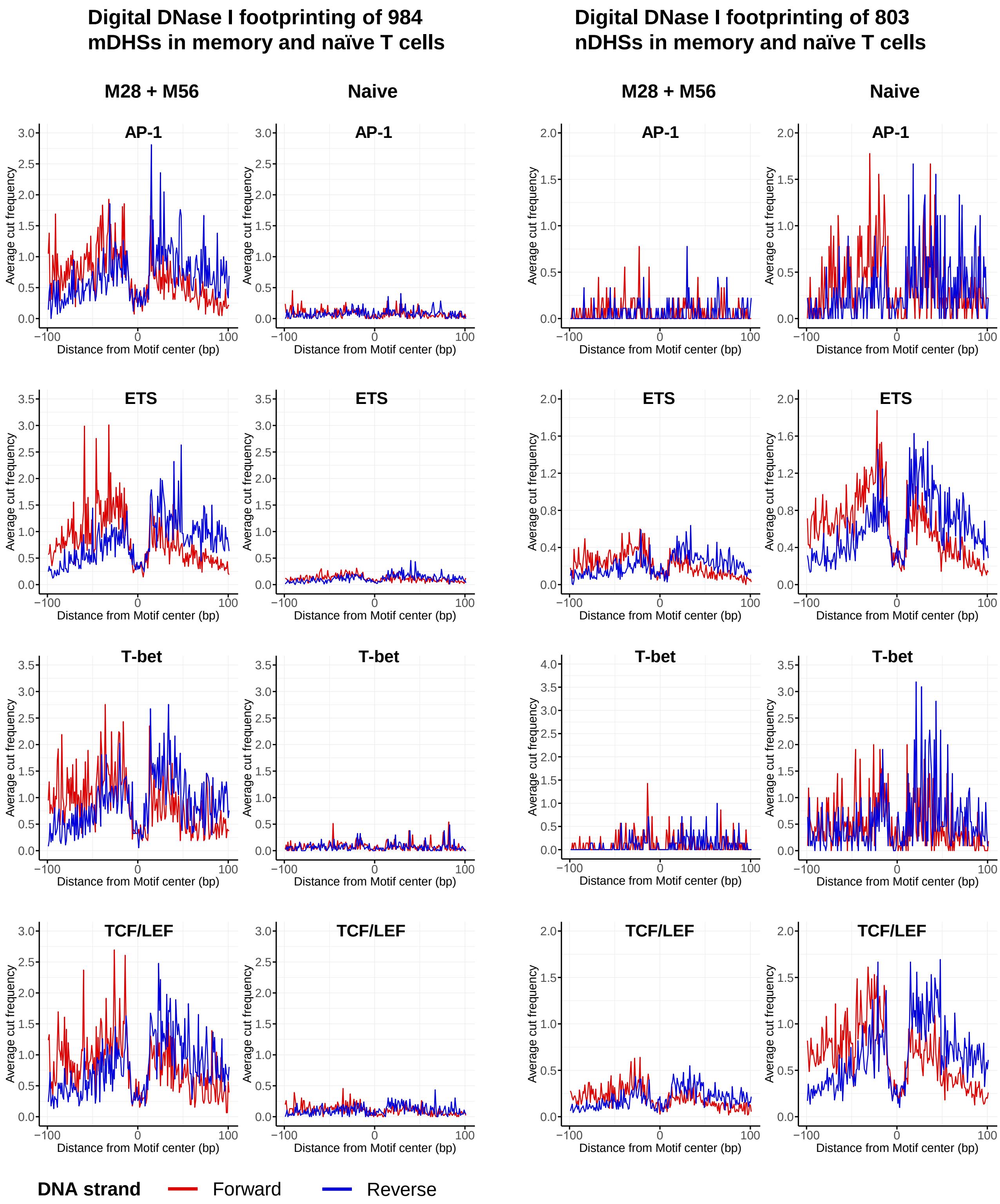

Figure S3

***In silico* ATAC footprinting of occupied TF binding sites in TM cells.**

The Wellington algorithm was used plot the accumulated ATAC signal over TF motifs in TM and TN cells in mDHSs and nDHSs defined in M28 and N.

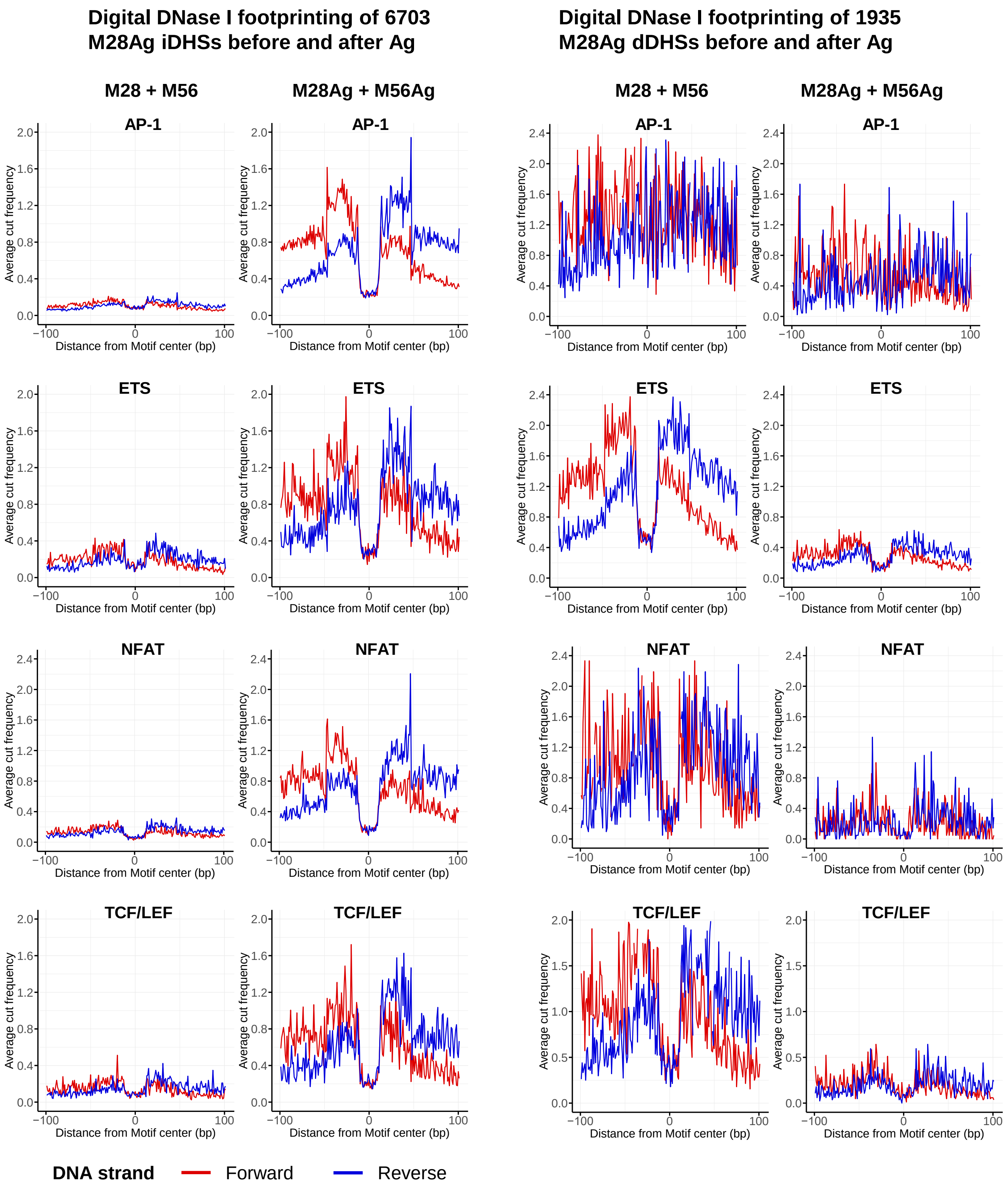

Figure S4

***In silico* ATAC footprinting of occupied TF binding sites in Ag-stimulated TM cells.**

The Wellington algorithm was used plot the accumulated ATAC signal over TF motifs in TM cells and TM + Ag in mDHSs and dDHSs defined in M28 and M28Ag.

Figure S5

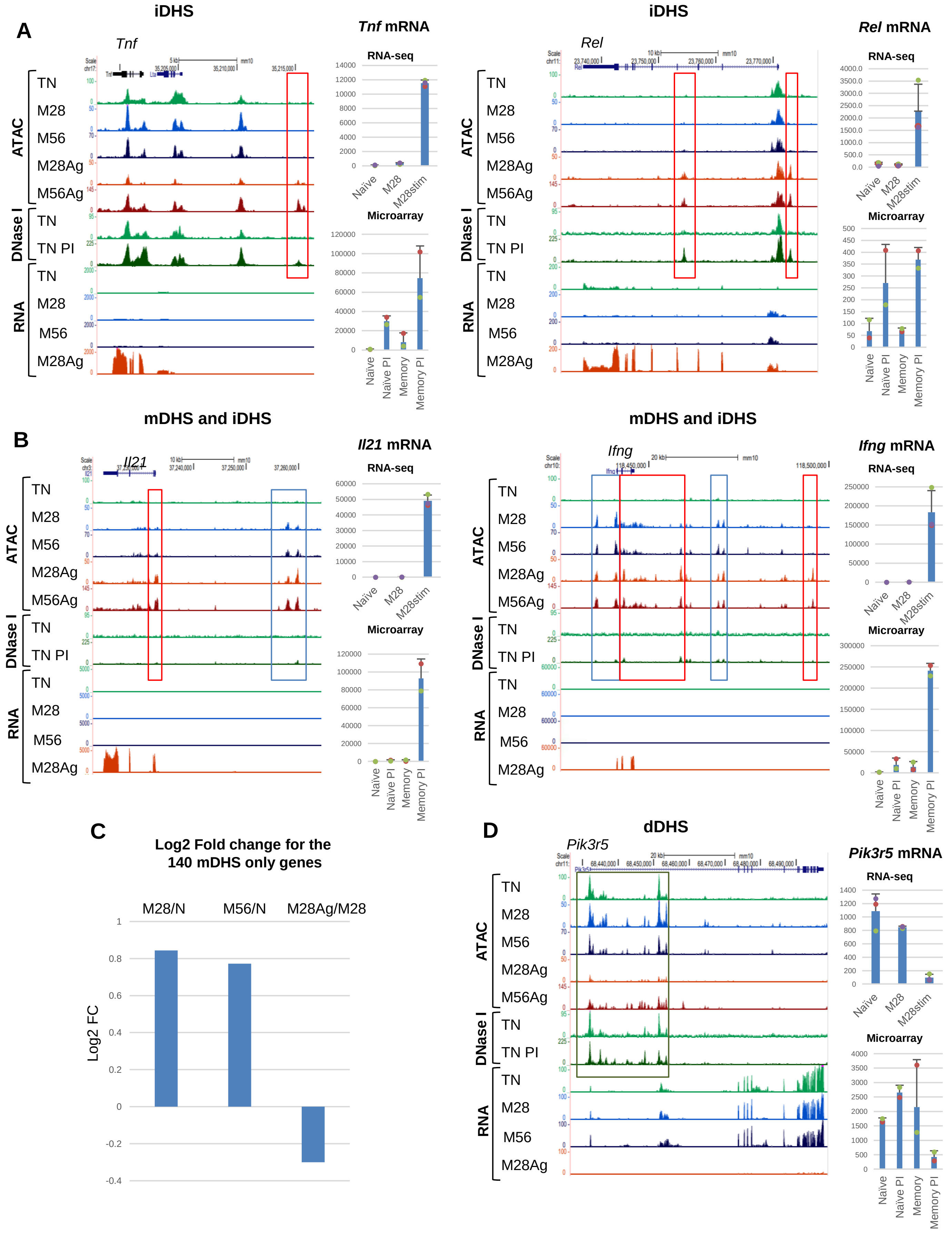
A
iDHS
Tnf
Tnf mRNA
RNA-seq
Microarray
ATAC
DNase I
RNA
TN
M28
M56
M28Ag
M56Ag
TN PI
Naïve
M28stim
Naïve PI
Memory
Memory PI
Rel
Rel mRNA
B
mDHS and iDHS
Il21
Il21 mRNA
Ifng
Ifng mRNA
C
Log2 Fold change for the 140 mDHS only genes
M28/N
M56/N
M28Ag/M28
Log2 FC
D
dDHS
Pik3r5
Pik3r5 mRNA

FIGURE S5.

Examples illustrating the roles of mDHSs, iDHSs and dDHSs.

(**A**, **B**, and **D**) UCSC browser screen shots showing ATAC-Seq, DNase-Seq and RNA-Seq data for representative genes with iDHSs (A), mDHSs and iDHSs (D), or dDHSs (D). Tracks include published DNase-Seq data for naïve CD4 T cells (TN) before and after PI-stimulation (3). Red boxes highlight iDHSs, blue boxes highlight mDHSs, and the dark green box highlights a dDHS. Shown at the right are RNA-Seq data for TN, M28 and M28 Ag (top) and published microarray data for responses to PI-stimulation of CD4 memory phenotype cells and naïve T cells (bottom) (3). Standard deviation is shown for 3 replicates for the RNA-seq data and 2 replicates for the microarray data. (**C**) Average Log2 values of the fold change (FC) in mRNA expression of genes associated with mDHSs in the indicated cell types.

Supplemental data files

Table S1

DHS subgroups defined by ATAC in TN and TM cells.

ATAC-seq data subsets from naïve T cells (TN), memory T cells (M7, M28 and M56) and Ag-stimulated memory T cells (M28Ag and M56Ag).

Table S2

KEGG pathway analyses of genes linked to specific subsets of ATAC peaks.

These subsets include (i) mDHSs identified in memory T cells in M7, M28 and M56, or in M7 only, (ii) genes in M28 which are linked to mDHSs but not iDHSs, are linked to mDHSs but not iDHSs, are linked to iDHSs but not mDHSs, or are linked to dDHSs but not mDHSs or iDHSs, and (ii) Tem and Tcm-specific DHSs.

Table S3

TN and TM -specific Gene subsets defined by RNA-seq.

RNA-seq data subsets from naïve T cells (TN), memory T cells (M7, M28 and M56) and Ag-stimulated memory T cells (M28Ag and M56Ag).

Table S4

Tem and Tcm specific subsets of mDHSs.

ATAC-seq data subsets for DHSs in Tem and Tcm-specific mDHSs from CXCR5+ve and CXCR5-ve memory T cells.
